# A Candidate Secreted Effector Protein of Rubber Tree Powdery Mildew Fungus Contributes to Infection by Regulating Plant ABA Biosynthesis

**DOI:** 10.3389/fmicb.2020.591387

**Published:** 2020-11-24

**Authors:** Xiao Li, Yuhan Liu, Qiguang He, Sipeng Li, Wenbo Liu, Chunhua Lin, Weiguo Miao

**Affiliations:** ^1^College of Plant Protection, Hainan University, Haikou, China; ^2^Key Laboratory of Green Prevention and Control of Tropical Plant Diseases and Pests, Ministry of Education, Hainan University, Haikou, China

**Keywords:** effector protein, powdery mildew fungus, rubber tree, ABA biosynthesis, plant immunity

## Abstract

Powdery mildew infects a wide range of crops and economic plants, causing substantial losses. Rubber trees (*Hevea brasiliensis*) are the primary source of natural rubber, and powdery mildew infection causes significant losses to natural rubber yields. How the causal agent, *Erysiphe quercicola*, establishes successful infection in rubber trees is largely unknown. Previously, 133 candidate secreted effector proteins (CSEPs) were identified in powdery mildew fungus. In this study, we characterize a CSEP named EqCSEP01276 for its function in suppressing host plant defense responses. We show that EqCSEP01276 is a secreted protein and is able to disturb the localization of 9-*cis*-epoxycarotenoid dioxygenase 5 (HbNCED5), a key enzyme in abscisic acid (ABA) biosynthesis in plant cell chloroplasts of *H*. *brasiliensis*. We also show that this effector inhibits ABA biosynthesis, and that in *H*. *brasiliensis* ABA is a positive regulator of the plant immune response against powdery mildew. Our study reveals a strategy by which powdery mildew fungus manipulates plant ABA-mediated defense for a successful infection.

## Introduction

Powdery mildew infects a wide range of agricultural, economic, and ornamental plants, causing significant losses. In rubber trees [*Hevea brasiliensis* (Willd. ex A.Juss.) Müll.Arg.], powdery mildew is one of the most severe diseases (Liyanage et al., 2017). The outbreak of powdery mildew in rubber trees severely threatens latex production since rubber trees are the primary source of latex, an important fundamental material for industry. *H*. *brasiliensis* is widely cultivated as a cash crop in tropical and sub-tropical areas of Asia and South America (Berthelot et al., 2016). The causal agent of powdery mildew disease was most recently identified as an obligate biotrophic fungus, *Erysiphe quercicola* (previously identified as *Oidium heveae*) (Limkaisang et al., 2005; Liyanage et al., 2017; Wu et al., 2019). It infects young leaves and other young tissues, causing leaf withering and defoliation (Liyanage et al., 2017). When conidiospores produced by *E*. *quercicola* make contact with the leaf surface, they germinate to form appressoria, which then breach the plant cell wall and allow the pathogen to enter the host. Subsequently, hyphae and lobe-shaped haustoria are produced inside the plant cells to acquire nutrients from the host. After 4 or 5 days, powdery mildew colonies consisting of a large number of vegetative hyphae and conidiospores are formed on the leaf surface (Limkaisang et al., 2005; Mei et al., 2016). Successful infection and colonization in the host also requires suppression of plant immunity, hence, *E*. *quercicola* can complete its life cycle in *Arabidopsis* mutants deficient in several crucial resistance-related genes (Mei et al., 2016). More recent genome analysis reveals that *E*. *quercicola* may have contracted gene families of carbohydrate metabolism and expended the repertoire of secreted effectors during evolution to facilitate host adaptation (Liang et al., 2018). Traditionally, preventing the outbreaks of rubber tree powdery mildew has largely been prevented by heavy application of brimstone as a fungicide, which causes soil and river water contamination (Mei et al., 2016). The molecular mechanisms of *E*. *quercicola* infection have been understudied, partly because of the inability to culture this pathogen in an artificial medium. This has made the development of suitable disease control strategies challenging.

Hosts and pathogens constantly compete against each other (Jones and Dangl, 2006; Dodds and Rathjen, 2010). Effectors must be secreted by plant pathogens into host cells to suppress immunity triggered by pathogen-associated molecular patterns (PAMPs). This layer of immunity is known as PAMP-triggered immunity (PTI). When secreted effectors are recognized by the host resistance gene products, another layer of immunity is triggered, known as effector-triggered immunity (ETI). However, effectors against resistance genes or ETI have evolved to sustain the life of pathogens (Dodds and Rathjen, 2010). Plant immunity often includes several defense responses such as the production of reactive oxygen species (ROS), pathogenesis-related protein accumulation, callose deposition, and hypersensitive response (HR) (Dodds and Rathjen, 2010; Kadota et al., 2015; Yamada et al., 2016). They function to inhibit pathogen hyphae growth and conidiation, or to kill microbe cells directly. Successful activation of plant immunity requires a complex signaling network that involves mitogen-activated protein kinase (MAPK) cascade, Ca^2+^-dependent pathway, and growth-controlling hormones such as salicylic acid (SA), jasmonate, and abscisic acid (ABA) (Vlot et al., 2009; Shigenaga et al., 2017; Zhang et al., 2018).

Abscisic acid has both positive and negative roles in plant immunity regulation depending on the type of pathogens, the tissues, growth stages, and the environment (Shigenaga et al., 2017). ABA induces nitric oxide (NO) accumulation and Ca^2+^ influx in guard cells, leading to stomatal closure and blocking bacterial penetration (Melotto et al., 2006). However, in *Arabidopsis*, mutants with defective ABA synthesis or perception showed increased resistance to *Pseudomonas syringae*, and ABA pretreatment increased susceptibility to the bacteria (de Torres-Zabala et al., 2007). In *H*. *brasiliensis*, foliar application of chitosan used as an elicitor enhances resistance to *Phytophthora* disease by inducing higher levels of ABA. Chitosan elevated the ABA levels in *H*. *brasiliensis* and increased the activity of the enzymes that catalyze ROS generation, such as catalase and peroxidase (Kuyyogsuy et al., 2018). It also induced high expression of defense-related enzyme genes, including *PR*-1, and heavy depositions of callose and lignin. Consistently, exogenous applications of ABA to *H*. *brasiliensis* caused effects similar to that of chitosan (Zhang et al., 2018). Additionally, the biosynthesis of ABA requires a key step the cleavage of *cis*-isomers of the ABA precursor xanthophylls by 9-*cis*-epoxycarotenoid dioxygenases (NCED) (Nambara and Marion-Poll, 2005; Frey et al., 2012). The NCED proteins are chloroplast-targeted, suggesting that the NCED-catalyzed cleavage reaction likely takes place in the chloroplast (Tan et al., 2003; Nambara and Marion-Poll, 2005). In *H*. *brasiliensis*, the elevation of ABA induced by chitosan application also induced the high expression of an *NCED* gene (*HbNCED*), consistent with NCED proteins being involved in ABA biosynthesis (Kuyyogsuy et al., 2018).

The *E*. *quercicola* genome contains 133 genes, encoding candidate secreted effector proteins (CSEPs) which only have homologs in other powdery mildew fungi (Liang et al., 2018). To understand the infection mechanisms of this pathogen, we investigated the potential functions of these CSEPs in the pathogenesis of *E*. *quercicola*. We found that successful infection of *E*. *quercicola* associates with suppression of host plant defenses, and that a CSEP labeled with sequencing number 01276 (EqCESP01276) (Supplementary Table 1) contributes to the suppression process. Further, analysis reveals that in *H*. *brasiliensis* ABA can induce a defense response against powdery mildew, and EqCSEP01276 inhibits ABA biosynthesis by targeting an NCED, HbNCED5.

## Materials and Methods

### Experimental Materials and Growth Conditions

*Nicotiana benthamiana* and the susceptible rubber tree cultivar, Reyan 7-33-97, were grown in a greenhouse with a 16/8 h light/dark cycle at 22°C. Bacterial material *Escherichia coli* DH5α was cultured on Luria–Bertani (LB) medium at 37°C. *Agrobacterium tumefaciens* GV3101 was cultured on LB medium at 28°C. The *Saccharomyces cerevisiae* YTK12 strain was cultured at 30°C in YPD medium. The powdery mildew pathogen *E*. *quercicola* (strain HO-73) was grown on rubber tree Reyan 7-33-97 plants.

### Functional Validation of Effector Signal Peptides

The signal peptide of EqCSEP01276 was cloned into the plasmid pSUC2 and transformed into the yeast strain YTK12 (Oh et al., 2009). Yeast cells were transformed with 0.5 mg of the individual pSUC2-derived plasmids using the lithium acetate method (Gietz, 2014). After transformation, yeast cells were plated on CMD minus Trp (CMD-W) plates (0.67% yeast nitrogen base without amino acids, 0.075% Trp DO supplement (Takara-Clontech, Japan), 2% sucrose, 0.1% glucose, and 2% agar) to confirm the transformation of the vector into the yeast strain. YPRAAA medium was used for assay for invertase secretion. Invertase enzymatic activity was detected by the reduction of 2,3,5-Triphenyl tetrazolium chloride (TTC) to an insoluble red-colored triphenylformazan. Transformants were cultured in liquid CMD-W medium for 24 h at 30°C. The pellet was collected, washed, and resuspended in distilled sterile water, and an aliquot was incubated at 37°C for 10 min. The supernatant was collected and placed into tubes containing 0.1% TTC solution. The color change was checked after a 5 min incubation at room temperature.

### Assays With Transient Expression of Proteins in *N*. *benthamiana*

To construct the EqCSEP01276^Δ*SP*^-GFP plasmid, EqCSEP01276^Δ*SP*^ cDNA was fused into the pBIN-GFP vector under the control of the *CaMV 35S* promoter. *Agrobacterium tumefaciens* GV3101 mediated transient expression of EqCSEP01276^Δ*SP*^-GFP in *N*. *benthamiana*. GV3101 was cultured in LB medium overnight with 50 mg/L rifampicin and 50 mg/L kanamycin. The cells were collected by centrifugation (3000 rpm, 3 min) and resuspended in 10 mM MgCl_2_. The suspensions were adjusted to an OD_600_ of 0.5. The leaves were infiltrated with EqCSEP01276^Δ*SP*^-GFP or GFP 24 h prior to infiltrating the same leaf areas with INF1 for PTI induction, and photographed after 3 or 4 days. GFP fluorescence was examined using a fluorescence microscope (Olympus BX51, Japan) at 48 h post-infiltration.

For the co-immunoprecipitation (co-IP) assay, EqCSEP01276^Δ*SP*^-GFP and HbNCED5-FLAG were co-expressed in *N*. *benthamiana* leaves; total leaf proteins were then extracted, followed by incubation of total proteins with anti-GFP-beads (ChromoTek, Germany) for 4 h. After incubation, the anti-GFP-beads were washed three times with washing buffer. Proteins bound to the anti-GFP-beads were boiled with 100 μL 10% SDS solution for 20 min to elute proteins that were detected with suitable antibodies (Abcam, United Kingdom).

### ROS Staining and Visualization of Callose Deposition

Accumulation of ROS was visualized by staining the leaves with 3,3′-diaminobenzidine (DAB). Rubber tree leaves were infiltrated with 0.1% DAB solution for 10 h in the dark and boiled in 95% ethanol for 30 min to remove chlorophyll. ROS accumulation was observed under a microscope and photographed (Dong and Chen, 2013).

For callose deposition visualization, rubber tree or *N*. *benthamiana* leaves were washed with double distilled water (ddH_2_O) and soaked in 95% ethanol, followed by boiling in water to clear the chlorophyll. Next, the transparent leaves were treated with staining buffer for 1 h in the dark at room temperature (0.1% aniline blue, 67 mmol/L Na_2_HPO_4_, pH 12.0) (Clay et al., 2009). Callose depositions were observed by fluorescence microscopy (Olympus BX51).

### Yeast Two-Hybrid Assays

The pGADT7 vector expresses proteins fused to amino acids of the GAL4 activation domain (AD). The pGBKT7 vector expresses proteins fused to amino acids of the GAL4 DNA binding domain (DNA-BD). The cells of Y2H Gold Yeast Strain were transformed with 0.5 mg of the individual plasmids using the lithium acetate method (Gietz, 2014). After transformation, yeast cells were plated on SD medium without Leu or Trp to confirm the transformation of the vector into the yeast strain. And SD medium without Leu, Trp, His, and Ade was used for assay to detect interaction. The positive control is a combination of pGADT7-T and pGBKT7-53. The pGADT7-T encodes a fusion of the simian virus 40 large T antigen and the GAL4 AD, and pGBKT7-53 encodes a fusion of the murine p53 protein and the GAL4 DNA BD. It has been reported that the murine p53 protein domain forms a complex with the 40 large T-antigen (Tan et al., 1986).

### Bimolecular Fluorescent Complimentary (BiFC) Assay

EqCSEP01276^Δ*SP*^ cDNA was fused into the sPYNE-N’YFP vector to generate the *35S*-EqCSEP01276^Δ*SP*^-N’YFP plasmid, and HbNCED5 cDNA was fused into the sPYCE-C’YFP vector to generate the *35S*- HbNCED5-C’YFP plasmid. Different combinations of plasmids were introduced into *N*. *benthamiana* leaf cells by GV3101-mediated transformation. Yellow fluorescent protein (YFP) fluorescence was examined using confocal microscopy (Leica TCS SP8, Germany).

### Chemiluminescence Measurement of ROS Production in *H*. *brasiliensis* Mesophyll Protoplasts

The transformation of *H*. *brasiliensis* mesophyll protoplasts was carried out and RNA was extracted as previously described (Zhang et al., 2016). For chemiluminescence detection of ROS production (Bellincampi et al., 2000), 10,000 protoplasts were suspended in a well of a 96-well plate containing 100 μL of luminol solution (200 μM luminol L-012, 10 μg/mL peroxidase, horseradish peroxidase). Relative fluorescence intensities of ROS samples treated with or without 10 μM (GlcNAC)_7_ were measured by a LB941 microplate reader (Berthold Technologies, Germany). Expression of genes of interest was confirmed by Reversed Transcript PCR (RT-PCR). The primer pairs used for RT-PCR were as follows: GFP-F (5’- ATGGTAGATCTGACTAGTCCTAGG-3’) and GFP-R (5’-CTTGTACAGCTCGTCCAT-3’), EqCSEP01276-F (5’-GGCCC GGTCGTCCGACGATCTA-3’) and EqCSEP01276-R (5’- TC AATTCTCATTTGTGTT-3’), HbActin-F (5’-CAGTGGTCGT ACAACTGGTAT-3’) and HbActin-R (5’-ATCCTCCAAT CCAGACACTGT-3’) (Zhang et al., 2016).

### ELISA for ABA Content Measurement

The ABA content in leaf tissues expressing CSEP01276, GFP, and wild-type was determined using an enzyme-linked immunosorbent assay (ELISA) kit (Agdia, United States) (Liu et al., 2014), as per the manufacturer’s instructions.

### Determination of HbNCED5 Quantity in Chloroplasts

Chloroplast and cytosolic fractions were isolated from 0.2 g *N*. *benthamiana* leaf tissue expressing HbNCED5-RFP and the proteins from chloroplasts and cytosol were extracted using the Minute-Chloroplast Isolation Kit (Invent Biotechnology, United States), according to the manufacturer’s protocol (Kanazawa et al., 2017). Total proteins were extracted from another 0.2 g leaf tissue expressing HbNCED5-RFP using Plant Total Protein Lysis Buffer (Sangon Biotech, China). From each sample, a 5 μg of protein was analyzed by SDS-PAGE and western blotting.

### Fluorescence Detection

GFP (emission wavelength: 488 nm, excitation wavelength: 680 nm), RFP (emission wavelength: 558 nm, excitation wavelength: 583 nm), chlorophyll (emission wavelength: 470 nm, excitation wavelength: 680 nm), YFP (emission wavelength: 513 nm, excitation wavelength: 527 nm), and aniline blue (emission wavelength: 665 nm, excitation wavelength: 600 nm) were detected. To examine fluorescence in *N*. *benthamiana* leaf cells using confocal microscopy (Leica TCS SP8, Germany), images of fluorescence channels were photographed using the Z-stack tool of confocal microscopy. When the Z-stack tool function is activated, multiple layers of one leaf area containing leaf epidemic and chlorophyll cells are scanned to generate each image.

## Results

### *E. quercicola* Is Able to Overcome PTI

**FIGURE 1:**
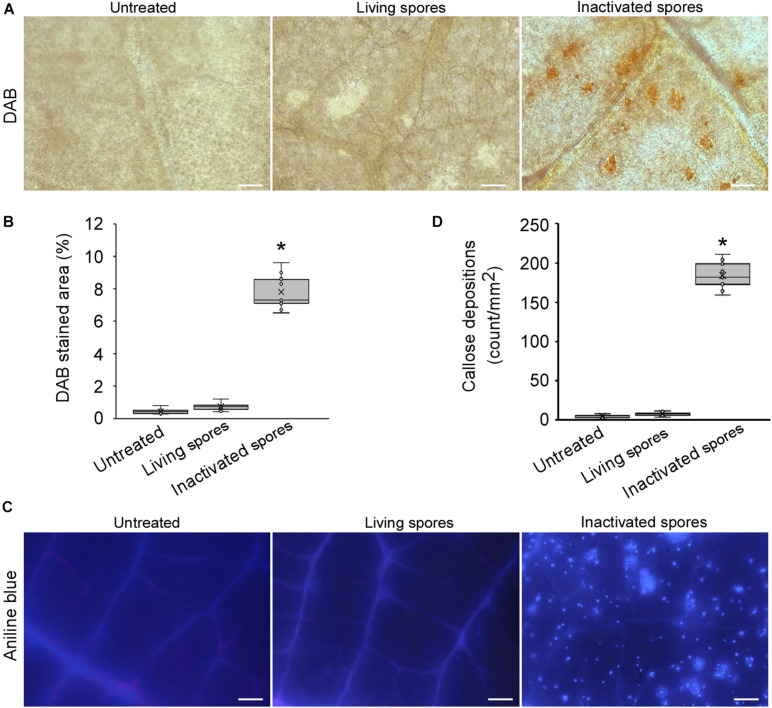
*Erysiphe quercicola* can suppress reactive oxygen species (ROS) burst and callose deposition in *Hevea brasiliensis*. **(A)** ROS accumulations in *H*. *brasiliensis* leaves inoculated with living and inactivated spores were detected by DAB in rubber tree leaves. The representative images were captured at 48 h post-inoculation. Bars = 50 μm. **(B)** The areas with ROS accumulations per 1 mm^2^ were analyzed with ImageJ software 1.49v. Three independent replicates with three areas per replicate were examined. Individual values (*n* = 9) are indicated by dots. Mean values are indicated by “×.” Median values are indicated by the middle line. Asterisks indicate significant differences (*P* < 0.01). **(C)** Callose depositions were visualized by aniline blue staining in *H*. *brasiliensis* leaves inoculated with living and inactivated spores. The representative images were captured at 48 h post-inoculation. Bars = 100 μm. **(D)** The numbers of callose spots per 1 mm^2^ area were analyzed with ImageJ software. Three independent replicates with three areas per replicate were examined. Individual values (*n* = 9) are indicated by dots. Mean values are indicated by “×.” Median values are indicated by the middle line. Asterisks indicate significant differences (*P* < 0.01).

The host immune response against powdery mildew was tested in *H*. *brasiliensis*. Live and inactivated spores (inactivated by boiling in water) of *E*. *quercicola* were suspended at a concentration of 1 × 10^6^/mL and sprayed on young leaves of *H*. *brasiliensis*. The inactivated spores can still provide PAMPs that can be recognized by the plant. At 48 h, ROS accumulation (as detected by DAB staining) was rarely observed at sites sprayed with living spores (Figures 1A,B). In contrast, ROS production was strongly induced at sites sprayed with inactivated spores. Moreover, a large number of callose depositions visualized by aniline blue staining were produced in leaves sprayed with inactivated spores but not with the living spores (Figures 1C,D). These results suggest that both ROS burst and callose formation are employed by the plant immune system against powdery mildew infection, and that *E*. *quercicola* can overcome this immune response.

### EqCSEP01276 Can Function to Suppress Plant Defense

**FIGURE 2:**
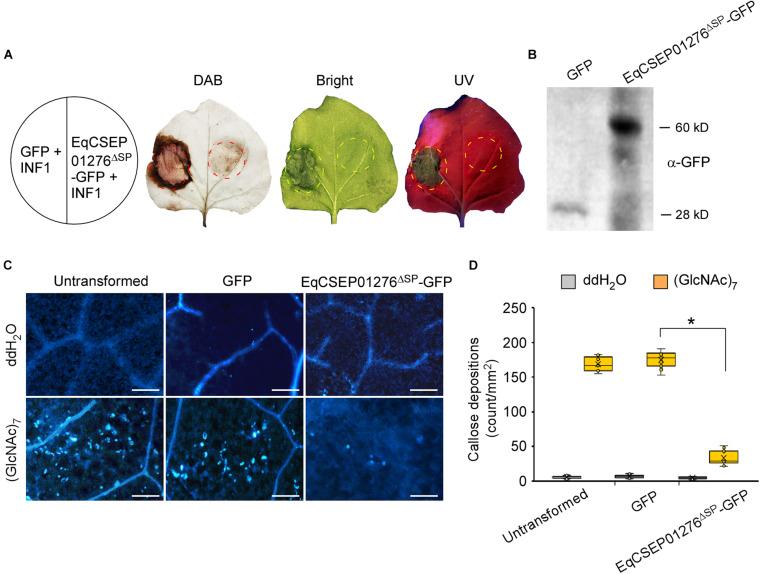
EqCSEP01276 expression inhibits defense responses in *Nicotiana benthamiana*. **(A)** GFP control and EqCSEP01276^Δ*SP*^-GFP were co-expressed with INF1 in *N. benthamiana* leaves. The infiltration into leaves for GFP and EqCSEP01276^Δ*SP*^-GFP expressions conducted 24 h prior to the infiltration for INF1 expression. DAB staining was used to detect ROS accumulations. INF1-induced hypersensitive response was indicated by ultraviolet (UV) light. **(B)** Expressions of GFP and EqCSEP01276^Δ*SP*^-GFP in *N. benthamiana* leaves were confirmed by western blot analysis. **(C)** 10 μM (GlcNAc)_7_ was infiltrated into *N. benthamiana* leaves transiently expressing EqCSEP01276^Δ*SP*^-GFP and GFP. Callose depositions were visualized by aniline blue staining. The representative images were captured at 24 h post-infiltration. Bar = 100 μm. **(D)** The number of callose fluorescent spots per 1 mm^2^ area were analyzed using ImageJ software. Three independent replicates with three areas per replicate were examined. Individual values (*n* = 9) are indicated by dots. Mean values are indicated by “×.” Median values are indicated by the middle line. Asterisks indicate significant differences (*P* < 0.01).

**FIGURE 3:**
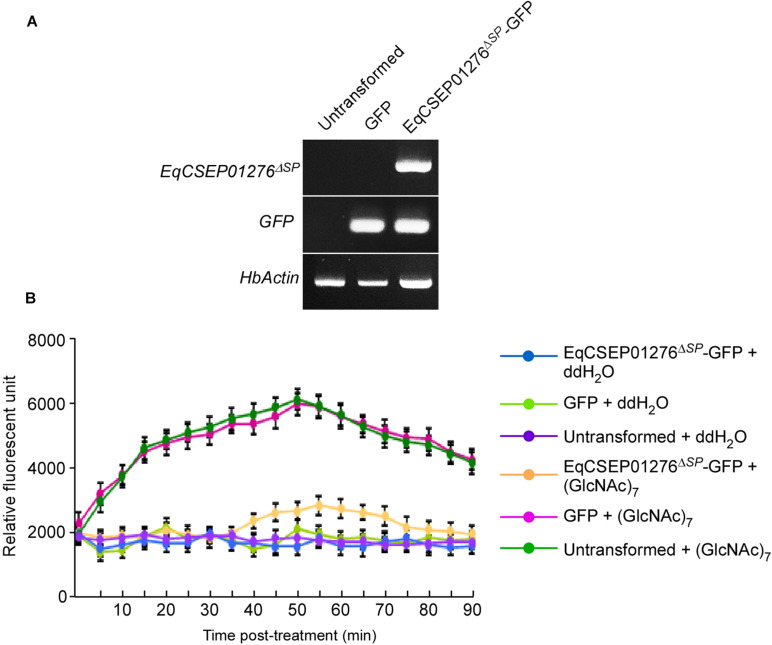
EqCSEP01276 suppressed reactive oxygen species (ROS) burst in *Hevea brasiliensis* mesophyll cell protoplasts. **(A)** RNA samples were extracted from *H*. *brasiliensis* mesophyll cell protoplasts with or without transformation and were analyzed by RT-PCR. *HbActin* was used as the reference gene. **(B)** Rubber tree mesophyll cell protoplasts were treated with 10 μM (GlcNAc)_7_ and fluorescence intensities of ROS labeled by Luminol were measured after treatment with (GlcNAc)_7_. Data presented are the means and SD values from three independent experiments.

We screened *E*. *quercicola* CSEPs that contribute to plant defense suppression using the dicotyledonous plant *N. benthamiana.* INF1, a *Phytophthora infestans* elicitor, was transiently expressed in *N*. *benthamiana* leaves by *A. tumefaciens*-mediated transformation to elicit ROS accumulation and HR (Kamoun et al., 1998). CSEPs were co-expressed with INF1 to test their effector function. Given that signal peptides are often thought to be cleaved from mature proteins (Owji et al., 2018), we used the form of effectors without signal peptides. Notably, *CaMV 35S* promoter-driven expression of a CSEP EqCSEP01276 with a signal peptide deletion and fused with a GFP-tag (EqCSEP01276^Δ*SP*^-GFP) significantly reduced the level of INF1-induced ROS accumulation compared to the GFP protein used as a control (Figures 2A,B and Supplementary Figure 1). Meanwhile, HR examined by ultraviolet (UV) light was inhibited by EqCSEP01276^Δ*SP*^-GFP expression (Figure 2A and Supplementary Figure 1). These results suggest that EqCSEP01276 can suppress INF1-induced defense responses.

(GlcNAc)_7_ is a derivative of chitin, generally known as a fungal PAMP. *N. benthamiana* leaves infiltrated with 10 μM (GlcNAc)_7_ formed a large number of callose depositions. However, we found that EqCSEP01276^Δ*SP*^-GFP expression in the leaves significantly inhibited callose deposition when compared with the GFP control or untransformed leaves (Figures 2C,D). Furthermore, we introduced vectors into mesophyll protoplasts derived from *H*. *brasiliensis* leaves expressing EqCSEP01276^Δ*SP*^-GFP because transient gene expression in *H*. *brasiliensis* protoplasts is feasible (Zhang et al., 2016). The expression of genes of interest was confirmed by RT-PCR analysis (Figure 3A). After 10 μM (GlcNAc)_7_ was applied, ROS burst was induced in the untransformed protoplasts and those that expressed only GFP as determined by the chemiluminescence detection assay (Bellincampi et al., 2000; Figure 3B). In contrast, EqCSEP01276^Δ*SP*^-GFP expression significantly inhibited ROS burst. Based on these observations, we concluded that EqCSEP01276 could disrupt the plant immune system.

### EqCSEP01276 Is a Secreted Protein

**FIGURE 4:**
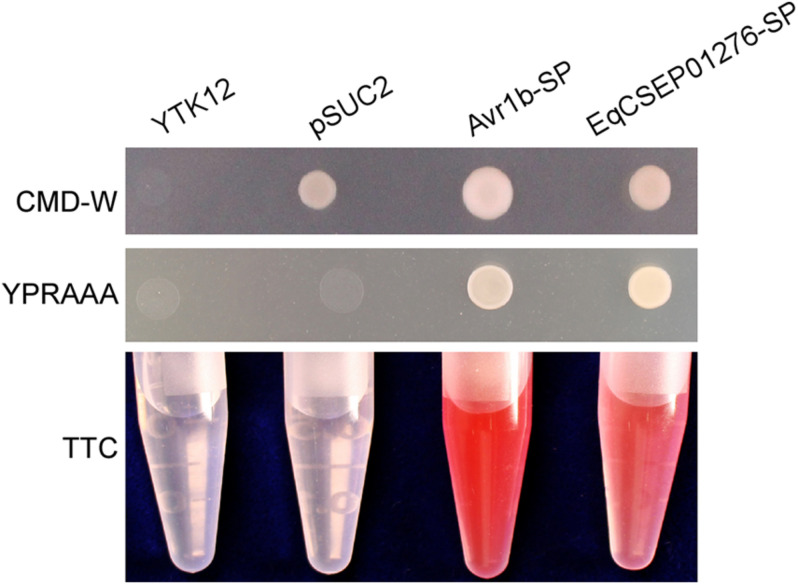
Functional validation of the signal peptide of EqCSEP01276 by the yeast invertase secretion assay. The EqCSEP01276 signal peptide was fused into the pSUC2 vector and was transformed into the yeast YTK12 strain. The predicted signal peptide of Avr1b was used as a positive control. The untransformed YTK12 and YTK12 carrying the pSUC2 vector were used as negative controls. Yeast growth on CMD-W (minus Trp) medium confirmed that the vector had transformed into the yeast strain. The growth on YPRAAA medium and color change of TTC confirmed invertase secretion. SP, signal peptide.

NCBI BLASTp analysis^1^ showed that EqCSEP01276 has only one homolog of EqCSEP01276 in powdery mildew, *Oidium neolycopersici* (Supplementary Figure 2). Thus, this CSEP probably has a specific role in the adaptation of *E*. *quercicola* to the host. Protein sequence analysis using SMART analysis service^2^ indicated that EqCSEP01276 carries a predicted N-terminal signal peptide with 17 amino acids (aa 1–17) and a t-SNARE region (aa 46–113), which are thought to guide protein secretion. We assayed the secretory function of the EqCSEP01276 signal peptide using the yeast secretion system. The YTK12 strain was transformed with the reconstructed pSUC2 vector to express the signal peptide of EqCSEP01276. The signal peptide of a known secreted protein, Avr1b, was used as a positive control. Untransformed YTK12 and empty pSUC2 vectors were used as negative controls. Similar to the Avr1b signal peptide, the EqCSEP01276 signal peptide, but not the empty vector, allowed YTK12 to grow on the YPRAAA medium (Figure 4). The strain transformed with the EqCSEP01276 signal peptide displayed secreted invertase enzyme activity that catalyzed the reduction of TTC to a red-colored compound (Figure 4). These results suggest that EqCSEP01276 is a secreted protein, and its signal peptide is required for its secretion.

### EqCSEP01276 Localization in Plant Cells

**FIGURE 5:**
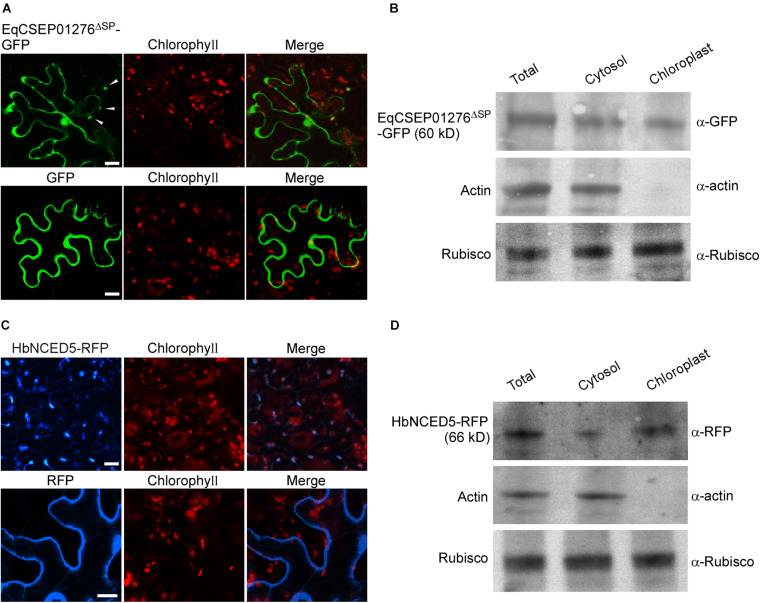
Localizations of EqCSEP01276 and HbNCED5 in plant cells. **(A)** Localizations of EqCSEP01276^Δ*SP*^-GFP and GFP in *N. benthamiana* leaves were examined by confocal microscopy. Each image of GFP or chlorophyll channel was photographed by using Z-stack tool of confocal fluorescence microscopy to scan one leaf area containing epidermal and mesophyll cells. White arrows indicate the chloroplast-localized EqCSEP01276^Δ*SP*^. Bars = 10 μm. **(B)** The total leaf proteins and proteins from cytosol and chloroplasts were extracted from *N. benthamiana* leaves expressing EqCSEP01276^Δ*SP*^-GFP. The resulting proteins were then analyzed by western blot using anti-GFP, anti-RFP, anti-β-actin, and anti-Rubisco antibodies. **(C)** Localizations of HbNCED5-RFP and RFP in *N. benthamiana* leaves were examined by confocal microscopy. Each image of RFP or chlorophyll channel was photographed by using Z-stack tool of confocal fluorescence microscopy to scan one leaf area containing epidermal and mesophyll cells. Bars = 10 μm. **(D)** The total leaf proteins and proteins from cytosol and chloroplasts were extracted from *N. benthamiana* leaves expressing HbNCED5-RFP. The resulting proteins were then analyzed by western blot using anti-GFP, anti-RFP, anti-β-actin, and anti-Rubisco antibodies.

We investigated EqCSEP01276 localization in plant cells. We transiently expressed EqCSEP01276^Δ*SP*^-GFP and GFP was used as control in *N*. *benthamiana* cells, and it was found that EqCSEP01276^Δ*SP*^-GFP was primarily distributed in the cytoplasm, displaying a similarity to GFP (Figure 5A). Additionally, some EqCSEP01276^Δ*SP*^-GFP signals were present in chloroplasts. We further validated the localization of EqCSEP01276^Δ*SP*^ in chloroplasts by western blot analysis (Figure 5B). In this assay, the proteins in chloroplasts were separately extracted from *N*. *benthamiana* leaves expressing EqCSEP01276^Δ*SP*^-GFP. The proteins in the cytosol and total leaves were also extracted. Actin was used to detect cytosolic protein contamination, and ribulose-1,5-bisphosphate carboxylase/oxygenase (RuBisCo) was used as the loading control.

### EqCSEP01276 Can Interact With HbNCED5, a Chloroplast-Localized Protein

**FIGURE 6:**
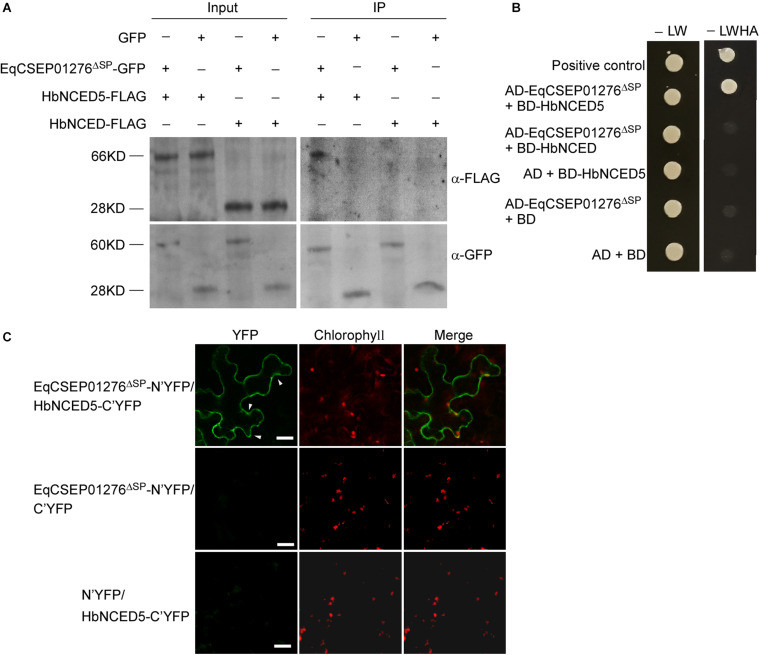
The interaction between EqCSEP01276 and HbNCED5. **(A)** Co-immunoprecipitation for interaction between EqCSEP01276^Δ*SP*^ and HbNCED5 or HbNCED. The total proteins from *N. benthamiana* leaves transiently expressing proteins of interest (Input) and the proteins eluted from anti-GFP beads were analyzed by western blot assay with anti-GFP or anti-FLAG antibodies. The GFP was used as the control. The sizes of protein bands: HbNCED5 (66 KD), HbNCED (28 KD), EqCSEP01276^Δ^ ^*SP*^-GFP (60 KD), GFP (28 KD). **(B)** In yeast two-hybrid assay, yeast transformants expressing EqCSEP01276^Δ*SP*^ and HbNCED5 or HbNCED were assayed for growth on SD-LW or SD-LWHA. L, leucine; W, tryptophan; H, histidine; A, adenine. The combination of AD-T and BD-53 was used as a positive control, and the other sets were used as negative controls. **(C)** Bimolecular fluorescent complimentary (BiFC) assay of the interaction between EqCSEP01276^Δ*SP*^ and HbNCED5. YFP signals were detected in the *N. benthamiana* leaves transiently co-expressing EqCSEP01276^Δ*SP*^-N’YFP and HbNCED5-C’YFP using confocal microscopy. The other sets were used as negative controls. Each image of YFP or chlorophyll channel was photographed by using Z-stack tool of confocal fluorescence microscopy to scan one leaf area containing epidermal and mesophyll cells. White arrows indicate the chloroplast-localized YFP. Bar = 10 μm.

To further investigate how EqCSEP01276 functions in plant cells, we identified EqCSEP01276 potential targets of the host plant. We conducted pull-down assays with purified EqCSEP01276-GFP from *N*. *benthamiana* leaves and protein extracts from *H*. *brasiliensis* leaves. We precipitated the proteins bound to EqCSEP01276 using anti-GFP beads and identified them using LC-MS/MS analysis. Meanwhile, GFP alone was used as a control to remove the non-specific proteins bound to the GFP-tag. The results indicated that the interacting proteins of EqCSEP01276 included HbNCED5, one of the key enzymes in ABA biosynthesis, and two other uncharacterized proteins (Supplementary Table 2). To confirm this interaction, full-size HbCED5 was used for tests. EqCSEP01276^Δ*SP*^-GFP and HbNCED5-FLAG were co-expressed in *N*. *benthamiana* leaves and co-immunoprecipitation was conducted using anti-GFP beads. This assay indicated that EqCSEP01276^Δ*SP*^-GFP but not GFP can interact with HbNCED5-FLAG (Figure 6A). The yeast two-hybrid assay was also conducted with HbNCED5 as bait and EqCSEP01276 as prey. The yeast strains transformed with HbNCED5 and EqCSEP01276^Δ*SP*^ showed growth in SD medium without Leu, Trp, His, and Ade (Figure 6B), suggesting an interaction between the two proteins. In addition, the interaction of the two proteins in *N*. *benthamiana* leaf cells was validated by the bimolecular fluorescent complimentary (BiFC) method. The YFP signals were present in the cytosol and chloroplasts (Figure 6C). Moreover, we did not detect an interaction between EqCSEP01276 and full-size HbNCED (accession: MF375917.1), another NCED protein from *H*. *brasiliensis*, by co-immunoprecipitation and the yeast two hybrid assay (Figures 6A,B), implying the specificity of the interaction between EqCSEP01276 and HbNCED5.

HbNCED5 contains a putative N-terminal chloroplast transit peptide (aa 1–49), which was analyzed by ChloroP 1.1 Server^3^ (Supplementary Table 3), and HbNCED5 probably has a chloroplast localization. We expressed HbNCED5 fused with an RFP tag (HbNCED5-RFP) in *N*. *benthamiana* leaves to analyze its localization. We found that HbNCED5-RFP displayed a punctate pattern of distribution (Figure 5C) different only from RFP, which was evenly distributed in the cytosol, as previously described (Lee et al., 2008). We further extracted chloroplast proteins from leaves expressing HbNCED5-RFP and detected the presence of HbNCED5-RFP in chloroplasts by western blotting (Figure 5D). These results indicate that HbNCED5 is a chloroplast-targeted protein.

### ABA Positively Regulates Plant Defense in *H*. *brasiliensis*

**FIGURE 7:**
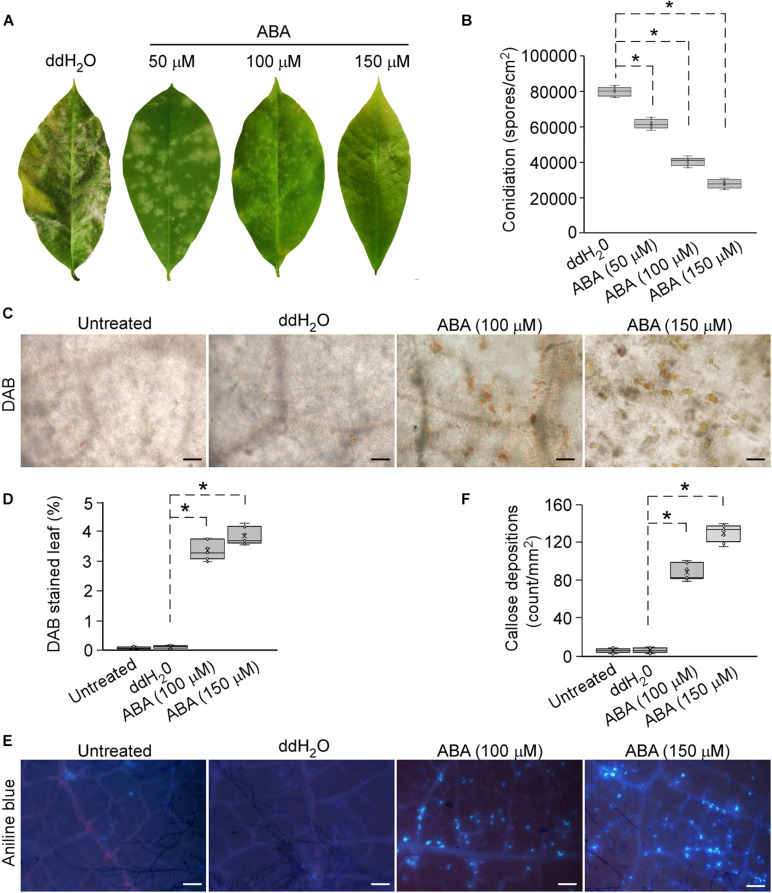
Exogenous application of abscisic acid (ABA) enhanced resistance against powdery mildew in *Hevea brasiliensis*. **(A)** Spraying ABA solutions (50, 100, and 150 μM) onto *H*. *brasiliensis* leaves, followed by inoculation of leaves with *E. quercicola* spores. The photographs were taken at 7 days post-inoculation. Three independent replicates with 10 leaves per replicate were examined. **(B)** The production of spores on *H*. *brasiliensis* leaves was quantified. Three independent replicates with three inoculated leaves per replicate were examined. Individual values (*n* = 9) are indicated by dots. Mean values are indicated by “×.” Median values are indicated by the middle line. Asterisks indicate significant differences (*P* < 0.01). **(C)** Reactive oxygen species (ROS) accumulations in infected leaves with or without treatment of H_2_O or ABA were detected by DAB staining. The representative images were captured at 7 days post-inoculation. Bars = 50 μm. **(D)** The areas of ROS per 1 mm^2^ in leaves were analyzed using ImageJ software. Three independent replicates with three areas per replicate were examined. Individual values (*n* = 9) are indicated by dots. Mean values are indicated by “×.” Median values are indicated by the middle line. Asterisks indicate significant differences (*P* < 0.01). **(E)** Callose depositions were visualized by aniline blue in infected leaves with or without treatments of H_2_O or ABA. The representative images were captured at 7 days post-inoculation. Bars = 100 μm. **(F)** The number of callose spots per 1 mm^2^ area was analyzed with ImageJ software. Three independent replicates with three areas per replicate were examined. Individual values (*n* = 9) are indicated by dots. Mean values are indicated by “×.” Median values are indicated by the middle line. Asterisks indicate significant differences (*P* < 0.01).

The interaction between EqCSEP01276 and HbNCED5 led us to suspect that EqCSEP01276 affects ABA biosynthesis. We investigated whether ABA contributes to the plant defense system against *E*. *quercicola* in *H*. *brasiliensis*. The young *H*. *brasiliensis* leaves with an exogenous treatment of ABA (50, 100, and 150 μM) or ddH_2_O (ABA solvent), were inoculated with *E*. *quercicola* spores (1 × 10^6^/mL). After 7 days, the leaves treated with 50, 100, and 150 μM ABA displayed enhanced resistance to *E*. *quercicola* infection compared with those treated with ddH_2_O (Figure 7A). Additionally, ABA treatment inhibited *E*. *quercicola* growth and decreased its spore production by at least 50% (Figure 7B). Furthermore, in leaves inoculated with *E*. *quercicola* spores, treatments with ABA (but not ddH_2_O) intensely induced ROS accumulation (Figures 7C,D) and callose deposition (Figures 7E,F). Thus, we conclude that ABA can induce defense responses against *E*. *quercicola* infection.

### EqCSEP01276 Expression Inhibits ABA Biosynthesis in *N*. *benthamiana*

We found that callose deposition in *N. benthamiana* was induced by the infiltration of 100 and 150 μM ABA into the leaves (Supplementary Figures 3A,B). In addition, callose formation induced by 10 μM (GlcNAc)_7_ was severely inhibited when 100 μM tungstate, an inhibitor of ABA biosynthesis (Hansen and Grossmann, 2000), was infiltrated into leaves (Supplementary Figures 3A,B). Thus, ABA appears to be a positive regulator of the immune system in *N*. *benthamiana*.

**FIGURE 8:**
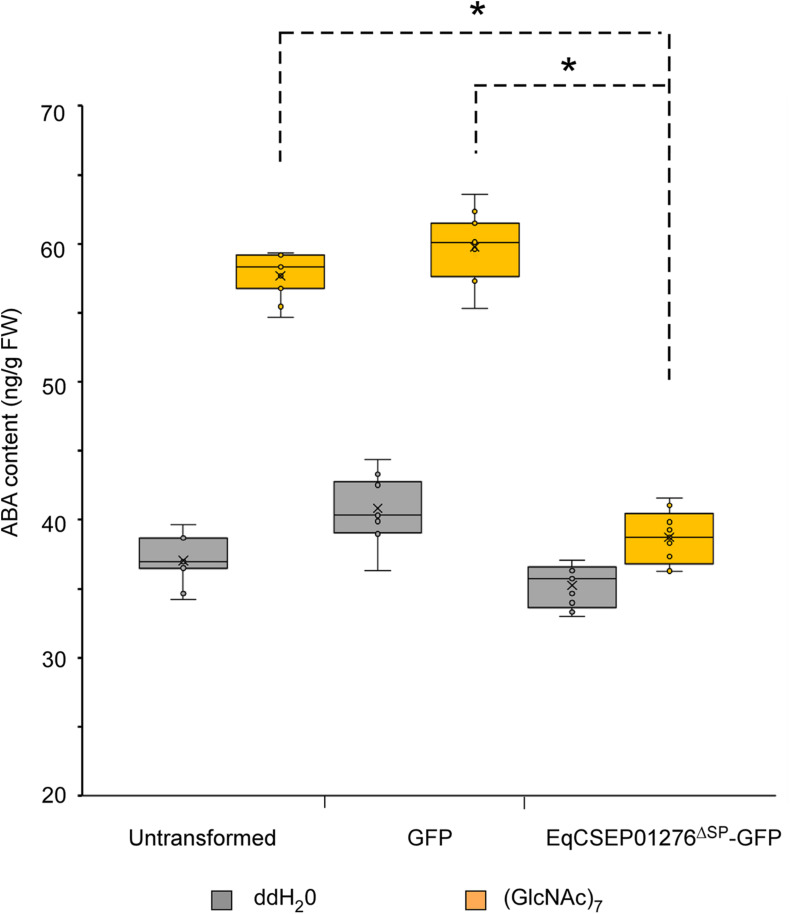
Determination of abscisic acid (ABA) content in *Nicotiana benthamiana* by ELISA. The ABA concentrations in *N. benthamiana* leaves treated with H_2_O and (GlcNAc)_7_. EqCSEP01276^Δ*SP*^-GFP expression suppressed the ABA content elevation induced by (GlcNAc)_7_. Individual values obtained from nine replicates are indicated by dots. Mean values are indicated by “×.” Median values are indicated by the middle line. Asterisks indicate significant differences (*P* < 0.01). FW, fresh weight.

We investigated whether EqCSEP01276 affects ABA biosynthesis using *N*. *benthamiana*. ABA concentration in leaf cells was determined by ELISA. The results showed that 10 μM (GlcNAc)_7_ induced ABA elevation (Figure 8), consistent with the positive role of ABA in the regulation of plant immunity. EqCSEP01276^Δ*SP*^-GFP expression significantly inhibited (GlcNAc)_7_-induced ABA elevation (Figure 8), suggesting that EqCSEP01276 functions in the downregulation of ABA biosynthesis.

### EqCSEP01276 Can Reduce the Amount of HbNCED5 in Chloroplasts

**FIGURE 9:**
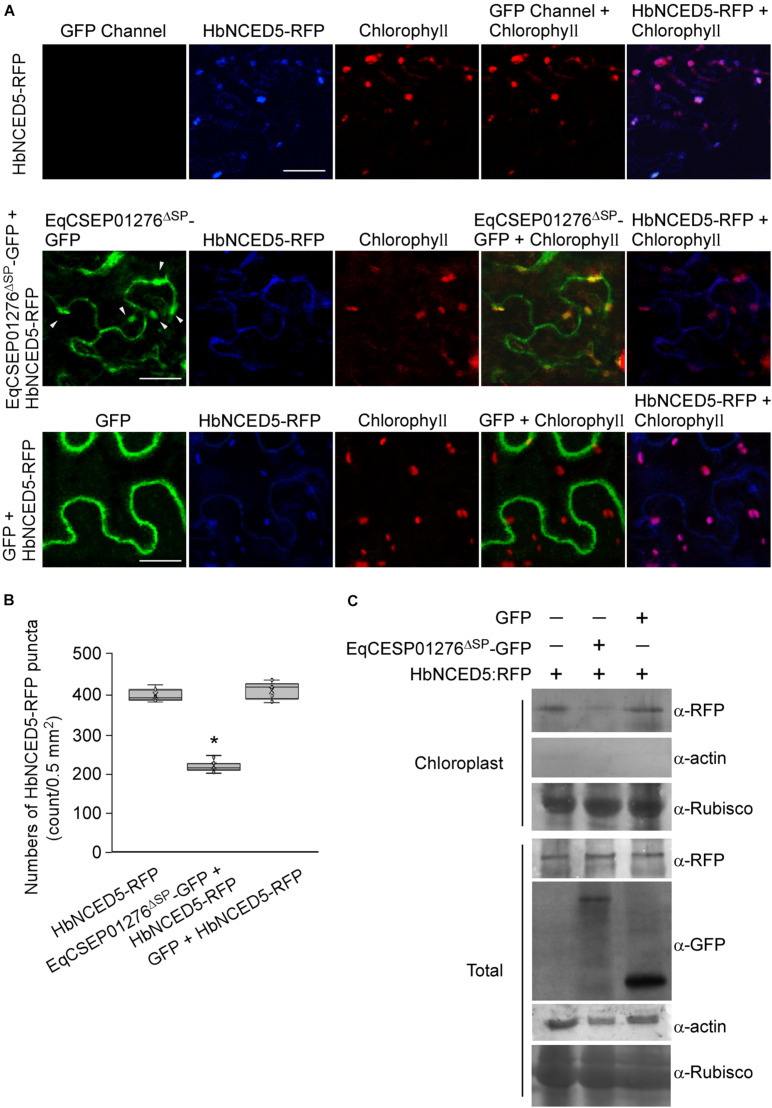
HbNCED5 localization in chloroplasts is altered by EqCSEP01276 expression in *Nicotiana benthamiana*. **(A)**. Co-expression of EqCSEP01276^Δ*SP*^-GFP and HbNCED5-RFP in leaves of *N. benthamiana* were examined by a fluorescence microscope. The other sets were used as controls. Each image of GFP, RFP, or chlorophyll channel was photographed by using the Z-stack tool of confocal fluorescence microscopy to scan one leaf area containing epidermal and mesophyll cells. White arrows indicate the chloroplast-localized EqCSEP01276^Δ*SP*^. Bar = 10 μm. **(B)** HbNCED5-RFP puncta per 0.5 mm^2^ area were analyzed with ImageJ software. Three independent replicates with three areas per replicate were examined. Individual values (*n* = 9) are indicated by dots. Mean values are indicated by “×.” Median values are indicated by the middle line. Asterisks indicate significant differences (*P* < 0.01). **(C)** Western blot analysis of total and chloroplast proteins extracted from *N. benthamiana* leaves expressing proteins of interest. The expression of EqCSEP01276^Δ*SP*^-GFP reduced HbNCED5-RFP abundance in chloroplasts. Asterisk indicates total protein used as positive control in analysis with anti-β-actin antibody.

We hypothesized that EqCSEP01276 interferes with HbNCED5 function in ABA biosynthesis. To test this, EqCSEP01276^Δ*SP*^-GFP and HbNCED5-RFP were co-expressed in *N*. *benthamiana* leaves and their intensities were examined using a fluorescence microscope. Co-expression of GFP and HbNCED5-RFP was used as a control. We observed that in cells in the presence of strong EqCSEP01276^Δ*SP*^-GFP signals, the intensity of HbNCED5-RFP with punctate distribution was reduced (Figures 9A,B). To confirm this further, chloroplast proteins extracted from leaves expressing the proteins of interest were subjected to western blot analysis. Consistent with our observation, EqCSEP01276^Δ*SP*^-GFP, but not GFP alone, significantly reduced the abundance of HbNCED5-RFP in chloroplasts (Figure 9C). We also noticed that the total amount of HbNCED5-RFP was not altered, both in the presence and absence of EqCSEP01276. Thus, our results imply that EqCSEP01276 probably perturbs the distribution of HbNCED5 in chloroplasts rather than promoting its degradation, resulting in failed ABA biosynthesis, which ultimately inhibits plant defense responses.

## Discussion

Powdery mildew is a devastating disease of rubber trees. Fundamental knowledge about its associated pathogen is urgently needed to develop advanced disease control techniques. In our study, we identified only one homolog of EqCSEP01276 in *O*. *neolycopersici*, which suggests a highly specific role of EqCSEP01276 in *E*. *quercicola* infection. Consistent with this, EqCSEP01276 was found to affect plant PTI by manipulating ABA biosynthesis in the host plants. EqCSEP01276 can target chloroplasts, which are the primary sites of photosynthesis, biosynthesis of plant hormones, production of ROS, and Ca^2+^-dependent signaling (Apel and Hirt, 2004; Lu and Yao, 2018).

In powdery mildew fungi, how CSEPs function in the infection stage remains uncharacterized, except for a few of CSEPs from cereal powdery mildew fungi. Our study provides more evidence that effectors of powdery mildew fungi are involved in PTI suppression. Some CSEPs from barley and wheat powdery mildew fungi can target plant immunity-associated proteins, including stress related small heat shock protein chaperones (HSP16.9 and HSP17.5) (Ahmed et al., 2015) and pathogen-related proteins (PR5 and PR10) (Pennington et al., 2016, 2019). A CSEP SVRPM3^*A*1/F1^ is a suppressor of AvrPm3_*a*__2__/f__2_-PM3A/3F recognition, which mediated race-specific resistance of wheat (Bourras et al., 2015). PM3A and PM3F are nucleotide-binding leucine-rich repeat receptor (NLR) immune proteins. A barley powdery mildew effector CSEP0064 functions as a ribonuclease-like protein to degrade plant ribosomal RNA (Pennington et al., 2019). Moreover, some cereal powdery mildew CSEPs can be recognized by cereal plant NLR proteins, including MLA1, MLA13, PM2, and PM3F/3A, and can induce ETI (Bourras et al., 2015; Lu et al., 2016; Praz et al., 2017).

A recent study reported that an effector secreted by the wheat stripe rust fungus, a biotrophic pathogen, can enter host chloroplasts and interact with a component of the cytochrome b6-f complex to inhibit photosynthesis and chloroplast-derived ROS (Xu et al., 2019). Additionally, bacterial pathogens also secrete numerous effectors by the type III secretion system to target host plant plastids, including chloroplasts. Well-studied examples of these effectors are HopN1 and HopI1 (Jelenska et al., 2007; Rodriguez-Herva et al., 2012). These studies, along with ours, recognize host chloroplasts as the main targets of biotrophic and hemibiotrophic pathogens.

We demonstrated that after entry into the host plant cells, EqCSEP01276 interacts with chloroplast-targeted HbNCED5 and reduces the HbNCED5 protein amount in the chloroplasts. We also noticed that the reduced amount of HbNCED5 in the chloroplasts is not a result of degradation initiated by CSEP01276 because the total amount of HbNCED5 is unaltered in leaf cells infiltrated with EqCSEP01276. Several effectors from other species of pathogens directly degrade plant target proteins. For example, the effector AvrPiz-t, secreted by rice blast fungus, targets two rice E3 ligases required for PTI and promotes their degradation via the 26S proteasome (Park et al., 2012, 2016).

Even though EqCSEP01276 can target the chloroplasts as HbNCED5, a putative chloroplast transit peptide is not present in EqCSEP01276^Δ*SP*^ (Supplementary Table 3). We assumed that this effector may adhere to the outer membrane of the chloroplast and cannot be efficiently transported into the chloroplast. Consistently, in our assays a large portion of EqCSEP01276 is not localized to chloroplasts and is different from HbNCED5, which contains a chloroplast transit peptide. The importation of proteins into chloroplasts is facilitated by a series of TOC receptors, such as Toc159 or Toc33/34, which are bound to the outer envelope membrane of the chloroplast and are exposed to the cytoplasm (Demarsy et al., 2014; Nakai, 2018), and after proteins are recognized by Toc receptors, they are transported into chloroplast via a channel formed by Toc75 (Nakai, 2018). Our results show that in plant cells EqCSEP01276 prevents HbNCED5 from targeting chloroplasts but does not reduce the total amount of HbNCED5. We reason that EqCSEP01276 intervenes with recognition to HbNCED5 by TOC receptors, causing an inhibition effect on importation of HbNCED5 into chloroplasts.

There are five NCED proteins in *H*. *brasiliensis* and they display high amino acid similarity to each other (Supplementary Figure 4). The specificity of the interaction between EqCSEP01276 and HbNCED5 led us to suspect that in HbNCED5 some amino acids or motifs, which are not present in other *H*. *brasiliensis* NCED proteins, probably have important roles in the interaction between EqCSEP01276 and HbNCED5. Moreover, it is possible that the abundances of those NCED proteins are different in *H*. *brasiliensis* leaf cells and may affect the opportunities of interaction with other proteins.

We showed that foliar application of ABA in *H*. *brasiliensis* inhibited powdery mildew conidiation and growth and increased callose depositions in *N*. *benthamiana*. These findings further support our observations that ABA signaling plays an important role in resistance. We also noticed that ABA is supposed to be an antagonist of SA biosynthesis, which positively regulates systematic resistance to biotrophic pathogens. Although the mechanism of antagonism between ABA and SA in plant resistance is not well defined, some key regulators of SA signaling, such as NPR1, are found to be affected by other hormones. Thus, the sharing of signaling proteins possibly connects different hormone pathways (Spoel et al., 2003, 2009; Tada et al., 2008). We hypothesize that elaborate systems have evolved to coordinate the two hormone pathways that may function at different time-points in pathogen infection. After ABA takes effect, ABA signaling is rapidly downregulated so that its inhibition of SA-signaling is removed. Moreover, we assume that the role of ABA in immunity may vary according to the types of PAMPs recognized by the plants. Future efforts to assess this hypothesis are warranted.

Overall, our study provides an insight into the biotrophic strategy by which rubber tree powdery mildew fungus modulates plant hormone-dependent resistance.

## Data Availability Statement

The original contributions presented in the study are included in the article/Supplementary Material, further inquiries can be directed to the corresponding author.

## Author Contributions

WM and XL planned and designed the research. XL, YL, QH, SL, WL, and CL performed the experiments. XL, YL, and WM analyzed the data and wrote the manuscript. All authors contributed to the article and approved the submitted version.

## Conflict of Interest

The authors declare that the research was conducted in the absence of any commercial or financial relationships that could be construed as a potential conflict of interest.

## Footnotes

1 https://blast.ncbi.nlm.nih.gov/Blast.cgi

2 http://smart.embl.de

3 http://www.cbs.dtu.dk/services/ChloroP

## Supplementary Material

Supplementary Figure 1 Determinations of ROS accumulation and HR in *Nicotiana benthamiana* leaves.

Supplementary Figure 2 The result generated by BLASTp analysis of EqCSEP01276 sequence.

Supplementary Figure 3 ABA contributes to plant defense in *Nicotiana benthamiana*.

Supplementary Figure 4 Sequence alignment of NCED proteins in *Hevea brasiliensis.*

Supplementary Table 1 EqCSEP01276 and HbNCED5 cDNA and amino acid sequences.

Supplementary Table 2 The *H. brasiliensis* proteins forming complex with EqCSEP01276-GFP.

Supplementary Table 3 The predicted results of chloroplast transit peptide (cTP) in proteins.
