# Supplementary material for: A Candidate Secreted Effector Protein of Rubber Tree Powdery Mildew Fungus Contributes to Infection by Regulating Plant ABA Biosynthesis
Source: Front Microbiol. 2020 Nov 24;11:591387. doi: 10.3389/fmicb.2020.591387 (PMC7721678; doi:10.3389/fmicb.2020.591387)
Supplement: Supplementary Figure 1 — Determinations of ROS accumulation and HR in Nicotiana benthamiana leaves. [file Data_Sheet_1.PDF]

Supplementary Materials

A candidate secreted effector protein of rubber tree powdery mildew fungus contributes to infection by regulating plant ABA biosynthesis

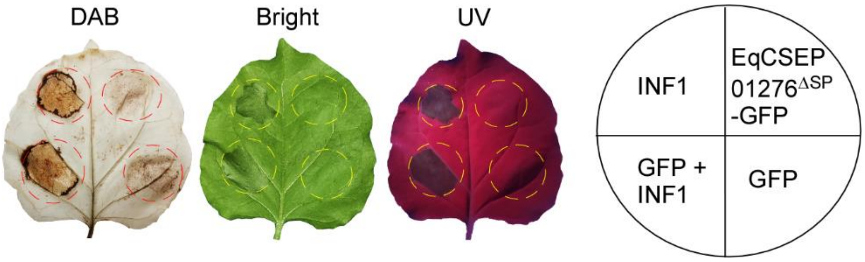

Supplementary Figure 1 Determinations of ROS accumulation and HR in *Nicotiana benthamiana* leaves.

DAB staining was used to detect ROS accumulations. INF1-induced HR was indicated by UV light. The circles label infiltration site.

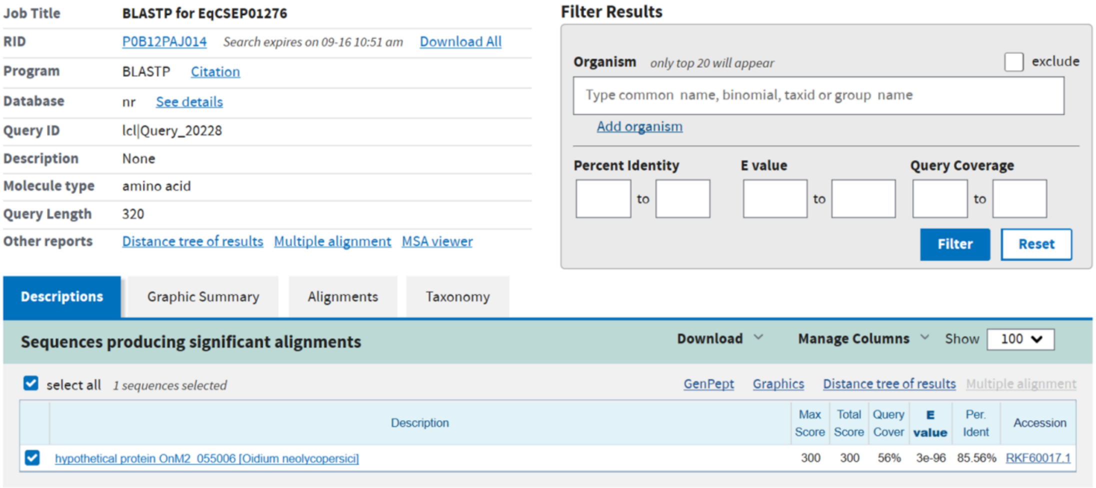

Supplementary Figure 2 The result generated by Blastp analysis of EqCSEP01276 sequence.

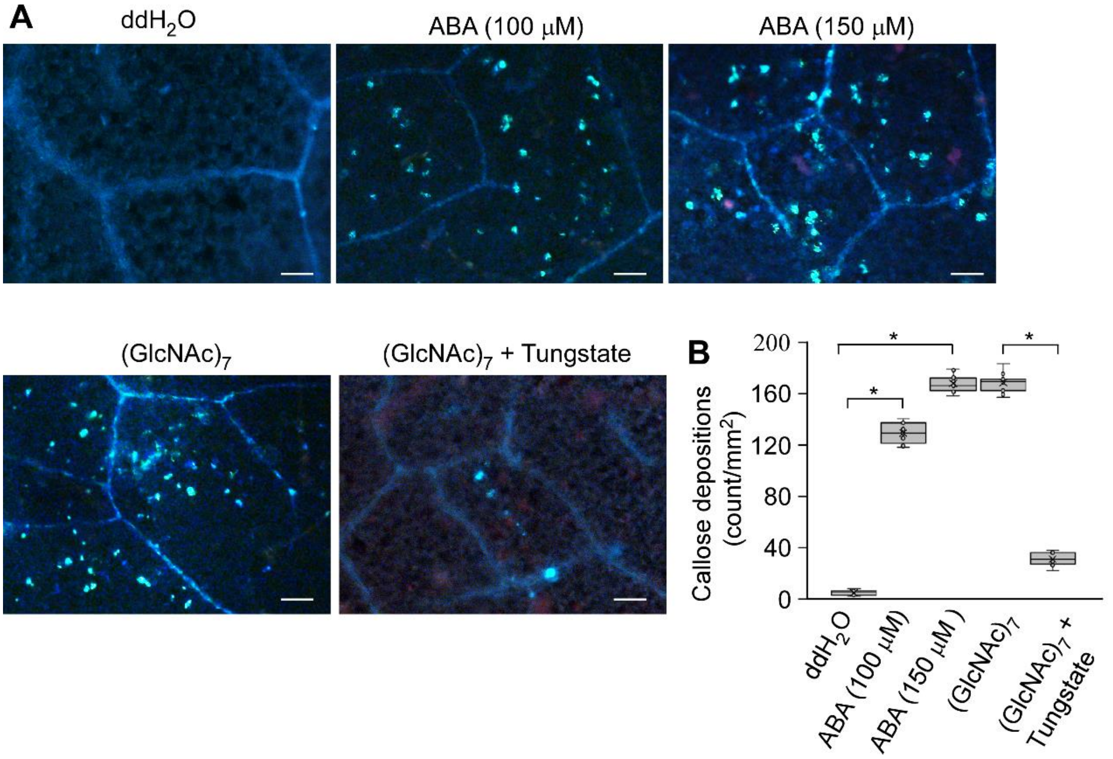

Supplementary Figure 3 ABA contributes to plant defense in *Nicotiana benthamiana*.

A. Callose depositions induced by ABA solution (100 and 150 μM in *N. benthamiana*. And tungstate solution (100 μM) suppress chitin-induced callose depositions. The representative images were captured at 24 h after treated with $(GlcNAc)_7$. Bars = 100 μm.

B. The numbers of callose spots per 1 mm^2 areas was analyzed with the ImageJ software. Three independent replicates with three areas per replicate were examined. Individual values (n = 9) are indicated by dots. Mean values are indicated by "×". Median values are indicated by the middle line. Asterisks indicate significant differences ($P < 0.01$).

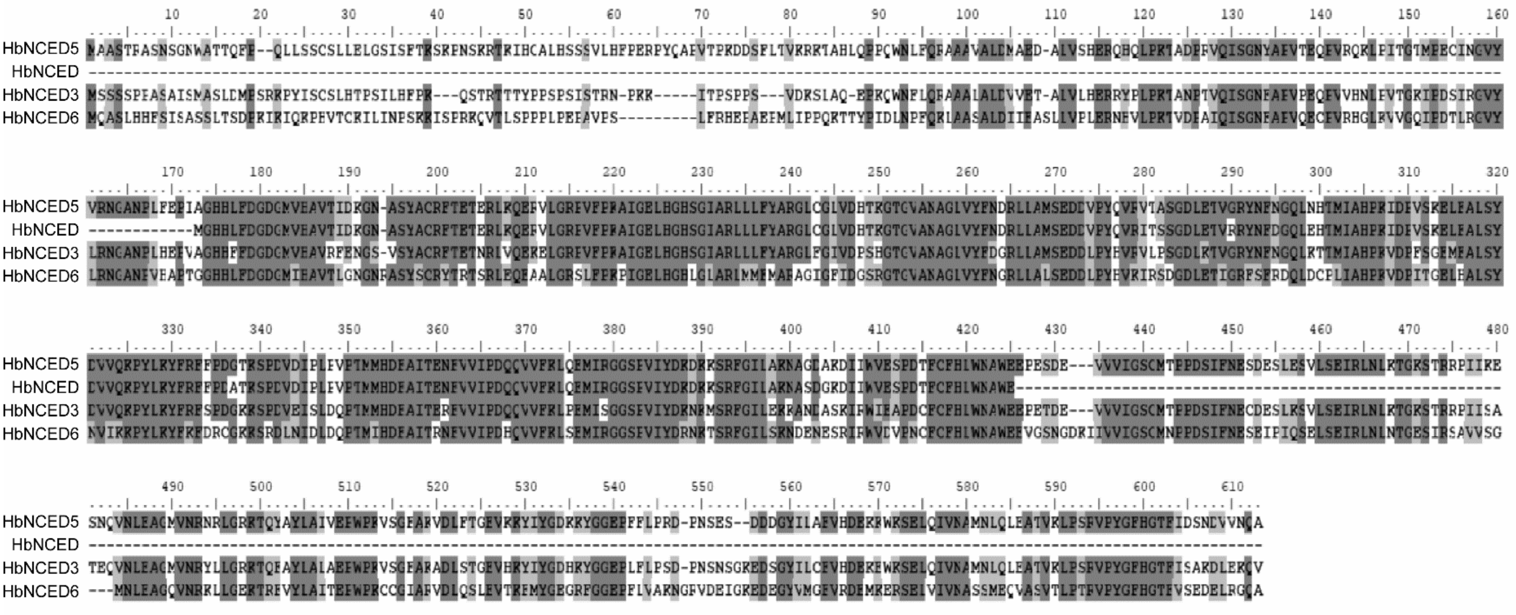

Supplementary Figure 4 Sequence alignment of NCED proteins in *Hevea brasiliensis*.
HbNCED5 (GenBank accession: XM_021834799.1), HbNCED (GeneBank accession: MF375917), HbNCED3 (GeneBank accession: XP_021661734.1), HbNCED6 (GeneBank accession: XP_021666736.1).

Supplementary Table 1 EqCSEP01276 and HbNCED5 cDNA and amino acid sequences.

EqCSEP01276 cDNA (GenBank accession: 2360406)	Length
ATGTCTGCCCTTCTCTTCTCACTTTTTACACTCTCTCAACTAGTAGCGTCGGGC<br>CCGGTCGTCCGACGATCTACTCTAGCAGCTGGCTGCGATCCCGCTACAAGAG<br>CTTTAGCCTTAGGAGTTCAAAATAATATAGCGGTGCAATATCAAGAGCTGTCC<br>ATATCAACTCAGCTGAGCAACACATTAGCAGCAAACCCAGTTGATGCAAATA<br>CCTTCCAAGTACAAAAGGCTCAACTATTATTTACAGTAGAACAAGGAATCGTG<br>ATTAGACAGAACAATCAAAACATCCCAAACTCAAACCAAGCAGTAATTCAGG<br>GCTTAAATACTGTGGCCAATGCTCAACAGCTAGAATTGCTGGCAACACAACA<br>GCTGGGAAATAGTGATCTAATCCAGACAGCTAATACAGTCAACAGCCTTATGG<br>CGGCATTTGCGGATGGGATCAACCAAAATTCGAATACTCTTACACAGGTTCTT<br>TCTCAATGTCCTGGTGGGCCATCCAACAATGGTTTAGGTACAACACAGCCACT<br>GCAATCTATTGTCCAAAATAATGGCAACAACTTAAACAGCGGCAACAACTTA<br>AACAACGGCAATAACATAAATAATGGCAATAATGGCAACAACATAAATAATGG<br>CAATAATGGCAATAATGGCAATAATGGCAACAATGGCAACAATGGCAACAAC<br>GGCAACAACGGCAACAACGGCAACAACGGCAACAACGGCAACAACGGCAA<br>CAATGGCAACAATGGCAACAACGGCAACAATGGCAACAACGAAAACAACGG<br>CATGAATGGGAACTCTCAATTTGGATCATTAAATGGTACAAATAGCACTATGG<br>CTTCTCCTACTAGCGCCTCAAACATTCAAGACGACAACTCACCATCTCCAACT<br>GGTTCCTCAAATAACAGAAATCAAAACTCGTCTTCTAGAAATGGTAGCTCAA<br>AAAACACAAATGAGAATTGA	963 bp
EqCSEP01276 amino acid sequence	Length
MSALLFSLFTLSQLVASGPVVRRSTLAAGCDPATRALALGVQNNIAVQYQELSIS<br>TQLSNTLAANPVDANTFQVQKAQLLFTVEQGIVIRQNNQNIPNSNQAVIQGLNT<br>VANAQQLELLATQQLGNSDLIQTANTVNSLMAAFADGINQNSNTLTQVLSQCPG<br>GPSNNGLGTTQPLQSIVQNNGNNLNSGNNLNNGNNINNGNNGNNINNGNNGN<br>NGNNGNNGNNGNNGNNGNNGNNGNNGNNGNNGNNGNNGNNENNGMN<br>GNSQFGSLNGTNSTMASPTSASNIQDDNSPSPTGSSNNRNQNSSSRNGSSKNTNE<br>N	320
HbNCED5 cDNA (GenBank accession: XM_021834799.1)	Length
ATGGCTGCATCTACTAGAGCCTCCAACTCCGGTAATTGGGCTACAACCCAATT<br>CCCTCAACTTCTTTCCTCTTGTTCTCTGCTGGAATTGGGCTCTATTTCCTTTAC<br>CAAAAGCAAGCCCAATAGTAAAAGAACCAAAATTCACTGCGCTCTACACTCC<br>TCCTCTGTTCTCCATTTCCCTGAACGGCCCTATCAAGCACCGGTAACTCCCAA<br>AGATGACTCCTTTCTTACTGTAAAACGCAAAACTGCCCACCTTCAACCGCCG<br>CAGTGGAACCTCTTTCAAAGAGCAGCAGCCGTAGCTTTGGACATGGCAGAAG<br>ATGCGTTGGTCTCTCACGAGCGCCAACACCAACTGCCCAAAACTGCTGATCC<br>AAGAGTCCAAATCTCCGGAAACTATGCTCCCGTGACTGAACAGCCCGTCCGG<br>CAGAAGCTTCCTATCACCGGAACTATGCCTGAATGCATTAACGGTGTCTATGT<br>GAGAAACGGTGCCAACCCACTTTTTGAGCCGATCGCCGGTCACCATTTATTTG<br>ACGGAGACGGCATGGTTCACGCTGTTACCATTGATAAAGGCAATGCAAGTTAT<br>GCTTGTCGTTTCACTGAAACAGAAAGGCTGAAACAAGAGAGAGTTTTGGGC<br>AGGCCAGTGTTTCCTAAAGCAATAGGCGAGCTACATGGCCACTCTGGTATAGC<br>AAGATTACTGCTTTTCTATGCCAGAGGATTATGTGGGCTCGTTGATCACACCA<br>AAGGAACTGGAGTGGCCAACGCCGGGCTTGTCTACTTCAATGACAGGCTTCT<br>TGCCATGTCTGAGGACGATGTTCCATACCAAGTGCGAGTCACTGCCAGTGGC<br>GATCTTGAAACTGTTGGCCGATACAATTTCAATGGCCAACTTAACCACACAAT<br>GATTGCCCACCCAAAAATTGATCCAGTTTCTAAGGAGCTATTTGCTCTGAGTT<br>ACGACGTCGTCCAAAAGCCGTACCTCAAGTACTTCCGATTCTTCCCCGATGGG<br>ACAAAATCACCGGATGTCGATATCCCTCTCCCAGTGCCAACCATGATGCATGA<br>TTTCGCTATCACTGAGAATTTCGTGGTGATACCTGACCAACAAGTCGTTTTCA<br>AGCTTCAAGAAATGATAAGAGGTGGCTCTCCGGTTATTTATGACAAGGACAA<br>GAAGTCACGGTTTGGGATTCTTGCAAAGAATGCTGGTGATGCTAAGGATATTA<br>TCTGGGTGGAATCACCGGACACTTTCTGCTTCCATTTATGGAATGCATGGGAG	1812 bp

GAGCCGGAATCCGATGAAGTAGTGGTAATCGGATCTTGCATGACTCCACCGG<br>ACTCCATCTTCAACGAAAGCGACGAGAGTTTAGAGAGCGTATTATCAGAAAT<br>CAGGCTCAATTTGAAGACGGGTAAGTCCACGCGCCGCCCCATAATTAAAGAA<br>TCAAACCAAGTGAATTTAGAGGCCGGGATGGTGAACAGGAACAGGCTGGGG<br>AGAAAGACTCAGTACGCTTACCTAGCCATTGTTGAGCCCTGGCCTAAGGTGT<br>CTGGTTTCGCCAAGGTGGATCTTTTTACAGGCGAGGTAAAGAAATATATATAC<br>GGCGATAAAAAATATGGTGGGGAGCCATTTTTCCTGCCAAGGGACCCTAATAG<br>CGAATCAGACGATGATGGGTACATTCTTGCTTTTGTCCATGACGAGAAGAAGT<br>GGAAATCGGAACTTCAAATTGTGAATGCCATGAATTTACAGTTAGAAGCCAC<br>GGTTAAGCTACCTTCCAGGGTTCCCTACGGGTTTCATGGCACATTCATTGACT<br>CAAACGACGTGGTGAATCAAGCATAG	
HbNCED5 amino acid sequence	Length
MAASTRASNSGNWATTQFPQLLSSCSLLELGSISFTKSKPNSKRTKIHCALHSSSV<br>LHFPERPYQAPVTPKDDSFLTVKRKTAHLQPPQWNLFQRAAAVALDMAEDALV<br>SHERQHQLPKTADPRVQISGNYAPVTEQPVRQKLPITGTMPECINGVYVRNGAN<br>PLFEPIAGHHLFDGDGMVHAVTIDKGNASYACRFTETERLKQERVLGRPVFPKAI<br>GELHGHSGIARLLLFYARGLCGLVDHTKGTGVANAGLVYFNDRLLAMSEDDVP<br>YQVRVTASGDLETVGRYNFNGQLNHTMIAHPKIDPVSKELFALSYDVVQKPYLK<br>YFRFFPDGTKSPDVDIPLPVPTMMHDFAITENFVVIPDQQVVFKLQEMIRGGSPVI<br>YDKDKKSRFGILAKNAGDAKDIIWVESPDTFCFHLWNAWEEPESDEVVVIGSCM<br>TPPDSIFNESDESLESVLSEIRLNLKTGKSTRRPIIKESNQVNLEAGMVNRNRLGR<br>KTQYAYLAIVEPWPKVSGFAKVDLFTGEVKKYIYGDKKYGGEPFFLPRDPNSES<br>DDDGYILAFVHDEKKWKSELQIVNAMNLQLEATVKLPSRVPYGFHGTFIDSNDV<br>VNQA	603

Supplementary Table 2 The *Hevea brasiliensis* proteins forming complex with EqCSEP01276-GFP.

**Putative name**	**Accession (Database: NCBI)**	**Number of identified peptides**
9-cis-epoxycarotenoid dioxygenase NCED5,	XM_021834799.1	3
hypothetical protein GH714_016523	KAF2310712.1	2
hypothetical protein kinase SD1-8	XP_021645696.1	2

Supplementary Table 3 The predicted results of chloroplast transit peptide (cTP) in proteins.

Sequence	Results generated by ChloroP 1.1 Server				
	Length	Score	cTP	CS-score	cTP-length
HbNCED5	603	0.550	Y	1.897	48
HbNCED	251	0.437	-	0.241	12
EqCSEP01276$^{\Delta SP}$	303	0.448	-	-2.450	83

"Y" means that the sequence is predicted to contain a cTP; "-" means that is predicted not to contain a cTP.
